# A Right to Know? Using Access to Information as Method in Critical Criminological Research

**DOI:** 10.1177/10778004241256140

**Published:** 2024-06-03

**Authors:** Brittany Mario, Jennifer Kilty

**Affiliations:** 1 Memorial University of Newfoundland, St. John’s, Canada; 2 University of Ottawa, Ontario, Canada

**Keywords:** access to information methods, critical criminology, government transparency, prison research, qualitative research, methodologies

## Abstract

Access to Information and Privacy (ATIP) requests are becoming an increasingly common method of qualitative inquiry, particularly for critical criminologists in Canada who face barriers in accessing Canadian prisons to conduct research. This article explores the politics of institutional gatekeeping and highlights the ongoing policing of critical criminological knowledge, necessitating the use of ATIP as a data collection method. Two case studies describe the strategies that the authors mobilized to acquire records from the Correctional Service of Canada (CSC) when their applications to conduct research inside prisons were denied. The authors argue that while access to information legislation is promoted as allowing for increased accountability and transparency of the government, real transparency is a public myth. This lack of transparency is linked to the ascendancy of administrative criminology in Canada, which ultimately devalues critical research and inhibits information flows in and out of carceral spaces.

## Introduction

Access to information and privacy (ATIP) requests are a growing methodological practice in critical social inquiry as they allow researchers to access the documents or “live archive” of otherwise inaccessible sites (Walby & Luscombe, 2017, 2019), what Goffman (1956–1959) describes as the “backstage work” of government officials and civil servants. In Canada, federal level ATIP^1^ requests are governed by the Access to Information Act (1985), which gives citizens, permanent residents, and any person or corporation in Canada a quasi-constitutional^2^ right to access the records of a government institution (Walby & Larsen, 2011b). The stated purpose of the Act is to make government information publicly available, to ensure that federal institutions in Canada are held accountable for their actions and decision-making, to foster greater transparency in public administration and government, and to “promote an open and democratic society and to enable public debate on the conduct of those institutions” (Access to Information Act, 1985, sec. 2(1)). Access to information requests thus facilitate studies in what Foucault (1991) describes as governmentality, or a critical analysis of the art of government.

Requesting government documents and information is a laudable research goal, as the material experience of doing so often stands in stark contrast to what is described in the Act. In this article, we examine these “materialities” and what they signify. Critical scholars stress that ATI methods are a powerful data collection tool when conducting research on policy decision-making and institutional practices (Piché & Walby, 2021; see also Luscombe & Walby, 2017; Piché, 2011, 2012; Walby & Larsen, 2011a; Walby & Luscombe, 2019). They observe that it is important to document and contest the barriers experienced when accessing information, as the often inevitable “messy process that went into accessing that data is often glossed over” (Walby & Luscombe, 2019, p.1). This article lays bare the messy processes of our respective ATIP experiences with one federal government agency, the Correctional Service of Canada (CSC), including seeking aid from the Offices of the Information Commissioner and the Privacy Commissioner to help us obtain the requested records.

While we concentrate our analysis on the Canadian prison context, given the similarities across different closed institutional sites (Foucault, 1980, 1991; Goffman, 1961), our analysis holds value for scholars working in other disciplinary fields, especially those attempting to access and research other total institutions (e.g., schools, hospitals), top-down bureaucracies (including but not limited to government agencies), or surveillance and security practices (Weber, 1968). Indeed, ATI scholars have used this method to seek access to data in a variety of different contexts and countries, such as to uncover the proliferation of unmanned aerial systems (drones) in Canada in relation to surveillance technologies (Bracken-Roche, 2019), in seeking “backstage” information from the U.K. border agency (Campbell, 2019), and obtaining information on the policing of protest behavior in the United States (Greenberg, 2019), to name but a few examples of many (see edited collection by Walby & Luscombe, 2019 for other applications of ATIP methods internationally).

We begin by outlining the state of carceral research access in Canada, taking care to detail the ways in which critical criminological knowledge is “policed” (Martel, 2004). We explore the politics of institutional gatekeeping with respect to seeking access to different kinds of criminological information to demonstrate the significance of utilizing ATIP as a data collection method. Next, we present two case studies that detail our respective attempts to secure: (a) government documents and (b) information that contextualizes why physical access to conduct research inside Canadian federal penitentiaries was denied. Our separate experiences were remarkably similar in that we were both denied access to conduct research inside federal prisons yet were ultimately “successful” when later seeking documents and information from the CSC, albeit with problematic delays. After outlining the details of the two cases, we move to contend that while access to information legislation is promoted as providing greater government accountability and transparency, real transparency is a “public myth” (Yeager, 2012) and that researchers must take on the responsibility of documenting their experiences of access denial, techniques of opacity, and information blockades to help provide some external oversight to these bureaucratic goals and responsibilities. We end with a discussion that situates the CSC’s lack of transparency, particularly in terms of research access denial, as part and parcel of the growth and power of administrative criminology in Canada, something Jock Young (1986) described as the discipline’s increased focus on the efficiency of the criminal justice system. Concentrating our analysis on the CSC’s approach to research, we suggest that the administrative criminologists working at this agency routinely devalue critical qualitative research on problematic methodological grounds that are only uncovered via the use of ATI methods.

## The Politics of Carceral Research Access and ATIP Requests in Canada

The CSC is not known for its transparency (Legault, 2010). Indeed, it is well known among critical prison scholars that access to information about carceral populations and practices is difficult to obtain and physical access to enter and conduct research in carceral sites is seldom granted (Kilty, 2014, in press; Hannah-Moffat, 2011; Martel, 2004; Mopas & Turnbull, 2011; Piché & Walby, 2021; Watson & van der Meulen, 2018; Wright et al., 2015; Yeager, 2012). As Wright et al. (2015) contend, “it comes as no surprise that the same walls that work to close prisoners within an institution also work to keep people out” (p. 113). To apply to conduct external research with the CSC, one must first complete a detailed application form, comprised of questions pertaining to: one’s professional qualifications; a review of the relevant literature; the research questions, design and methodology; and the impact of the proposed research on CSC resources, quotidian functions, and overall mission. Notably, researchers who are affiliated with a university must obtain approval from their institution’s Research Ethics Board (REB) prior to submitting an external research proposal to CSC. An executive committee at the national headquarters Research Branch then reviews the research proposal to “asses the anticipated benefits and relevance of the proposed research for CSC” ( CSC, 2017, sec. 2). According to the CSC’s (2017) Commissioner’s Directives regarding research, proposals are assessed based on several criteria, such as the contribution of the project to the achievement of CSC’s mission, the level of disruption the project may pose to quotidian functions, the quality of the methodology, researcher qualifications, the project’s anticipated benefits to CSC, and the level of risk posed by the project.

According to its website, the CSC welcomes collaborative research partnerships; in practice, however, this is rarely the case.^3^ Researchers who are perceived to be critical, or who “challenge the assumptions underpinning hegemonic correctional approaches, or whose research may bring the system into disrepute,” are routinely denied access to population information or to enter carceral institutions for research purposes (Hannah-Moffat, 2011, p. 446). There are different motivations to deny access (to information, documents, people, or physical sites) to external researchers, one of which is a political motivation to prevent knowledge acquisition that may be used to critique the institution, its actors, their practices, and institutional decision-making (Hannah-Moffat, 2011; Stevens, 2020; Watson, 2015; Wright et al., 2015). External researchers have also been denied access because the CSC’s research branch was conducting research on the same topic (Kilty, 2014; Hannah-Moffat, 2011). Still, in other cases, no justification for access denial is provided at all.

At the root of research access denial are the bureaucratic practices of institutional gatekeeping and protectionism (Hannah-Moffat, 2011). The CSC is concerned with reputation management and tends to favor positivist research methods and the production of research that aims to demonstrate efficient, evidence-based interventions, as opposed to exploratory or ethnographic qualitative research (Carlen, 2002; Hannah-Moffat, 2011; Martel, 2004; Watson, 2015). The CSC’s in-house research efforts, in conjunction with the very limited external research that is permitted, have come to define the kinds of information about Canada’s penal sites that are publicly available. Piché (2012) argues that preventing external researchers from accessing carceral institutions is a deliberate mechanism the CSC engages to control information flows and thus the kind of knowledge that is produced about their policies and practices. Most external researchers are therefore left to use ATIP methods^4^ and to acquire information through formerly incarcerated persons, including those on parole or other forms of community supervision, and other key informants and stakeholders about the inner workings of carceral settings (Watson, 2015).

Indeed, Martel (2004) memorably described this institutional impenetrability as one of the ways in which the CSC engages in the policing of criminological knowledge. In Martel’s (2004) case, the CSC aimed to discredit her research by claiming that qualitative research methods are overly subjective and, as such, are biased and untrustworthy. Indeed, she reports that the CSC alleged that prisoners’ “ways of knowing” were not legitimate forms of “scientific” data, which is demonstrative of a kind of testimonial and thus epistemic injustice (Fricker, 2007). By systematically dismissing insights gleaned from criminalized people, the CSC is attempting to preserve not only their institutional reputation, but also their institutional power. If correctional authorities position such insights as the naïve ramblings of untrustworthy or ignorant sources, they can also reject any critiques lobbed against institutional decision-making and practice (Foucault, 1991). In this way, the CSC policed criminological knowledge by banalizing qualitative methodologies and alternative (nonpositivist) epistemologies in an attempt to devalue Martel’s ground-breaking research on segregation practices in federal prisons for women and to dismiss the significance of the policy implications that her research identified.

To receive federal records through an ATIP request, one must apply directly to the government agency from which one is seeking information or material documents. The process for submitting a request varies by government agency—some entail online applications, while others require the applicant to send a hard copy request in the mail. These varied requirements reflect but one layer of bureaucracy that is involved in accessing information (Weber, 1968). Additional bureaucratic layers emerge when agencies establish gatekeeping practices to manage access to their records, including the creation of special divisions or dedicated personnel whose sole purpose is to assess requests and either grant or deny access to documents, information, and other institutional records (Larsen & Walby, 2012; Luscombe et al., 2017; Piché, 2012; Walby & Larsen, 2011a, 2011b; Weber, 1968). The steps involved in submitting a formal request for records to the CSC include: (1) retrieving available online information; (2) mailing a paper copy of a written request form; and (3) providing a $5 cheque to the ATIP office at CSC. Government agencies are required by law to provide written notice indicating whether they will grant access to the requested records and to provide all or part of the records within 30 days. Access to records is never guaranteed, but rather is

contingent on the intensity of “information management” in any government agency, the political contentiousness of the request, the limits of the Canadian ATI law and oversight mechanisms, and the complexities of requester-agency interactions and request wording. (Larsen & Walby, 2012, p. 17)

Although staff are assigned to evaluate ATIP requests, these individuals do not assist applicants to understand government language, nor do they identify what kinds of information or documents exist. In this sense, government knowledge remains a black box and applicants make requests through a certain degree of guesswork. Once submitted, requests are sent to the ATIP coordinator at the respective agency, who is an “arbitrator of state power and interests” within this political dynamic (Luscombe & Walby, 2017, p. 381). The ATIP coordinator is responsible for ensuring the records are gathered and for determining what will be released or withheld. This process reflects Weber’s (1968) classic treatise on bureaucracy, the ideal-typical characteristics of which include such things as hierarchical organization and regulation of all decision-making power, formal lines of authority, fixed areas of activity, rigid divisions of expertise and labor, continuous execution of assigned tasks, officials with expert training, and qualifications being evaluated by organizational rules not individuals (in our case, this was reflected in the CSC’s methodological bent toward positivism—discussed below).

Despite the government’s claims about transparency and accountability and of “making government accessible to everyone” (Government of Canada, 2023), accessing government records is wrought with challenges, barriers, politics, and constant negotiations between researcher and ATIP coordinator—implications of bureaucratization (Weber, 1968) that increase opacity rather than transparency. Indeed, “barriers to ATI abound” (Walby & Larsen, 2011b, p. 624), which is why this method, while growing in popularity, remains under-utilized (Walby & Luscombe, 2017, 2019; Yeager, 2012). In practice, ATIP requests reveal how the “mundane and bureaucratic workings of FOI are more multi-faceted and less linear” than any government agency request process might suggest (Luscombe & Walby, 2017, p. 379). In fact, ATIP and FOI requests are “more akin to a Pandora’s box insofar as [they] involve creatively handling unexpected barriers and setbacks” (Luscombe & Walby, 2017, p. 379).

To successfully receive government records, one must inevitably engage in what Larsen and Walby (2012; see also Walby & Larsen, 2011a, 2011b) refer to as “access brokering,” which is “the range of interactions involved in the filing and processing of an ATI request” and thus “an interactive, mediated process” (p. 17). The process involves “negotiation, contestation, and technological mediation” (Walby & Larsen, 2011b, p. 628) and, as our individual cases demonstrate, a significant degree of personal determination and perseverance by the requester when they are inevitably faced with delays, refusals, and other barriers. It is common for ATIP coordinators to ask the applicant to rephrase and to narrow the scope of their request in some way, although it is up to the applicant whether and how to do so. While narrowing one’s request may speed up the access process, doing so provides no guarantee that the records sought will be released.

Larsen and Walby (2012) describe such “techniques of opacity” as the various formal and informal manners through which government agencies structure—or prevent—the acquisition of records. We see techniques of opacity in the access to information law itself in the form of exemption and redaction clauses. Government agencies have the right to leave out entire sections of records or to redact information, leaving the requester with all but incomprehensible displaced words and phrases. For example, the second author supervised a Master of Arts student who submitted an ATIP request in 2016 for copies of offender complaints and grievances that spoke to health, illness, and aging related issues over a 5-year period. She received 500 pages of material that were so redacted that the displaced words across the pages amounted to little more than five pages of indecipherable text. While it is important to redact the names of people and institutional sites to maintain anonymity, to redact the material almost entirely rendered it completely useless. More than redaction, requests for government documents and information may be partially or fully refused if it is decided that the impact of the disclosure would unreasonably harm the operations of the state or violate the privacy of those named in the records (Gilbert, 2000).

Systemic delays are another technique of opacity, one that prevents researchers from timely access to requested materials (Larsen & Walby, 2012). Perpetual postponements are common and often push requests “beyond the statutory limits outlined in legislation and other guidelines” (Piché, 2012, p. 245). While applicants requesting Canadian federal government records generally do not incur exorbitant costs to acquire these records, those seeking provincial and records from jurisdictions around the world may encounter high fee estimates for searching, retrieving, reviewing, redacting, and scanning or photocopying records. These fees often act as a deterrent to requesting information, as applicants may be left with no choice but to abandon or drastically modify their requests due to fiscal restraints (Kingston et al., 2019; Luscombe & Walby, 2017; Piché, 2012). Piché (2012), for example, was given notice of a $5600 fee to acquire records from the Prince Edward Island Office of the Attorney General, which prompted him to significantly revise his query and ultimately resulted in a “radical disjuncture between the records initially sought and those received, although at a reduced cost of $275” (p. 245). Despite the reduction in cost, $275 may still be a financial barrier for some, particularly for graduate students who do not have research grants to fund such requests.

## The Two Case Studies

We take up Piché’s (2011) call for scholars to detail their experiences of trying to acquire government records through ATIP/FOI applications, outlining our separate—yet remarkably similar—experiences in submitting requests to the CSC for access to internal documents and information. Our respective research journeys each began with a request for external research access to conduct in-depth qualitative interviews with federal prisoners and various correctional staff members. Being denied access led the first author to shift the methodological design of her doctoral research project and to instead submit an ATIP request for internal government materials that would enable an alternate line of sight into her research object. For the second author, the purpose of the ATIP request was to uncover details that would better explain why her application to conduct research in federal penitentiaries was denied.

### Case 1—Accessing CSC Documents

My doctoral research explored correctional programming for federally sentenced women in Canada and deployed ATI methods to collect materials pertaining to correctional programs for women. In February 2018, after being denied access to conduct interviews with incarcerated women and correctional staff, I submitted a request to the CSC for access to program facilitator manuals, participant workbooks, and any policy guidelines that directed or impacted correctional programming. In the application, I listed all the relevant records I sought to acquire after researching CSC’s website for the types of documents that were available to request. I hoped that gaining access to these documents would “reveal the processes behind the creation of texts, allowing [me] to develop an understanding of the networks of agencies and chains of decisions that underlie official discourse” (Walby & Larsen, 2011b, p. 624).

On February 19, 2018, I received an email from the senior analyst at CSC’s ATIP division seeking clarification about the requested records. While I took this acknowledgment as a positive sign, such requests for clarification are not written notices that the records will be made available. I emailed the ATIP coordinator to check on the status of my request in May 2018, 2 months after the statutory limit of 30 days that government agencies have to respond to requests. I did not receive a reply. After a month of trying to make contact through email and phone calls with no response or acknowledgment, I submitted a formal complaint to the Office of the Information Commissioner (OIC) in June 2018. On June 26, an OIC investigator emailed to begin the formal complaint process.

Five months after CSC’s statutory 30-day due date, I had still not received any records. CSC claimed to have upward of 18,000 pages of material related to my request and asked if I would remove one set of documents (training manuals) to reduce the overall volume and make it easier for them to locate the records. I was uncertain about the content of these documents, so I rejected their request. On October 8, 2018, 9 months^5^ after I made the initial request, I followed up with the OIC investigator who identified that the CSC had provided a final disclosure date of December 21, 2018. Perhaps predictably, given the holiday season, I did not receive the records that December. It was not until January 30, 2019, that I received a partial disclosure (200 of the estimated 18,000 pages) of the material requested.

**Figure 1.**
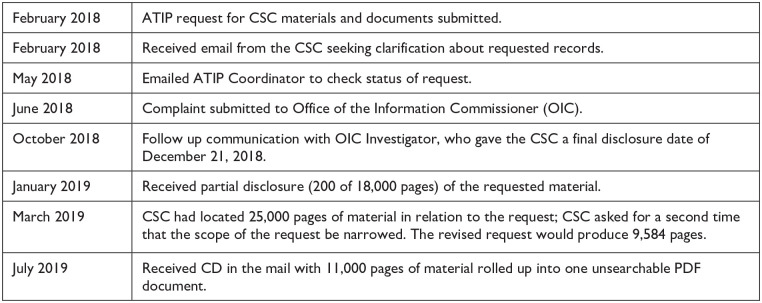
Case 1 CSC-ATIP Timeline.

On March 11, 2019, the OIC investigator disclosed that the CSC found my remaining request produced 25,000 pages of records; they once again asked me to narrow the number of requested documents. To expedite the process, I opted to narrow the list of 1200+ categories of records to 304 categories, which the ATIP office at CSC advised would reduce the page count to 9,584 and provided June 19, 2019 as the final disclosure date. Once again, I did not receive the disclosure package on time. After another delay and two mailing errors, I received the material by mail on July 25, 2019, 18 months after my original request. It came in the form of a CD containing 11,000 pages of federal programming documents, training manuals and materials, and institutional programming guidelines for incarcerated women which were compiled into one enormous and unsearchable PDF document.^6^ Figure 1 provides a visual summary of the researcher’s full ATIP timeline, from request to disclosure of records.

### Case 2—Accessing Prisons and the Criteria for Determining Access

In November 2018, I communicated via email with representatives of the CSC Research Branch, who agreed to an in-person meeting to discuss my project. In advance of this meeting, I sent a draft of my application for access to conduct research inside seven federal penitentiaries, including two Indigenous healing lodges (one for men, one for women). As scholars are routinely denied access because of the additional human resource costs that are required to manage external visitors to any carceral site, I initially thought to request access only to interview staff and had planned to interview former prisoners who now lived in the community; however, I was counseled to revise the application to include interviews with incarcerated people, having been instructed that once research access is granted, that there is no difference in terms of the expenditure of human resources. I was also advised to clarify the empirical value of the project particularly by identifying how it would practically benefit the CSC (more on this in the discussion). I submitted the revised application for research access in January 2019.

The project was designed to examine how emotions circulate in carceral space, the affective politics of incarceration, and how interpersonal interactions shape the feeling and expression of emotion in different spaces, with the goal of mapping the emotional geography and emotion cultures of federal penitentiaries. The project also aimed to examine the felt experience of two transformative policy shifts in correctional governance, namely how staff and prisoners felt about and experienced the new Structured Intervention Units (SIUs), the spaces designed to replace segregation after the courts deemed the practice unconstitutional, and the prison needle exchange pilot, designed to add a new feature of harm reduction to the federal correctional environment. Both policy shifts were controversial, and we speculate that my research team’s interest in them elicited red flags for correctional staff.

There is no information online as to the structure or timeline of the CSC’s external research access evaluation process, which turned out to be excessively long as it required consultation with the research branch sectors (e.g., Women Offender Research and Indigenous Research), regional Executive Committee (EXCOM) Sub-Committees, and the wardens of the carceral sites implicated in the project. Exemplifying bureaucratic opacity, slowness, and inefficiency, I was informed that I was being denied research access on February 11, 2020—14 months after requesting it. The email contained a general signature from the “Research Branch,” with no individual CSC representative signing off or leaving a name for future communication or appeal. The email simply noted that access was denied because “the current period of policy implementation and transition to new operational requirements are not conducive to the proposed research study.” As global lockdowns to curb COVID-19 transmission were instantiated 1 month later, I did little other than email the representatives I met with in December 2018 requesting clarification on the reasons for access denial, which went unanswered. In May 2021, I filed an ATIP request for the following information:

All correspondence and documentation (e.g., emails, letters, faxes, memos, meeting minutes, decision making criteria, briefing notes, presentation decks, information sheets, issue sheets, hand written notes, memorandums, terms of reference, evaluation criteria, phone or meeting transcripts, and agendas) regarding research application titled “X” submitted by X from the University of X Department of X to CSC EX-COM Subcommittee on Research and any other record holders that were consulted, including but not limited to the EX-COM Subcommittee on Women Offenders, EX-COM Subcommittee on Indigenous Offenders, regional subcommittees and institutions, including wardens and other regional and institutional coordinators.

**Figure 2.**
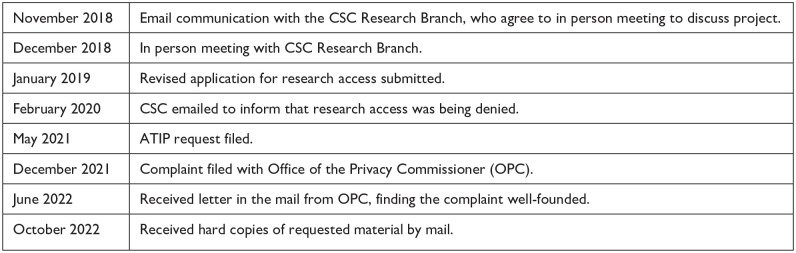
Case 2 CSC-ATIP Timeline.

The CSC did not provide these materials within the legislated time limit of 30 days and after significant delay, I contacted the Office of the Privacy Commissioner (OPC)^7^ on December 9, 2021, to file a complaint. I received a letter from the OPC dated June 29, 2022, finding the complaint “well-founded” and advising that if the CSC did not provide the requested information by October 21, 2022 (521 days after my initial request for it) that I should alert the OPC Acting Investigator to reopen the case. I received printed copies of the requested materials in the mail by that date.^8^ Notably, my own curriculum vitae and project application were printed repeatedly because they had been attached to various email communications; this content amounted to 160 of the 350 pages provided, or 46% of the material received. The rest of the material consisted of 16 pages (4.5%) of email memorandum service requests to various CSC actors (e.g., EXCOM subcommittees, regional representatives, wardens) to review the project proposal; 40 pages (11.4%) of internal CSC email—most of which spoke to arranging various meetings where discussion of the project proposal was on the agenda; 91 pages (26%) of my own email exchanges with CSC employees about the project; 6 pages (1.7%) of completely redacted CSC meeting agendas; 11 pages (3.1%) indicating material that was withheld due to the Privacy Act; 4 pages (1.1%) of unusably redacted handwritten notes; and only 22 pages (6.3%) of actual CSC employee commentary discussing the proposal. Given the lengthy delay in providing the requested material, that so little of the content was usable or relevant to the request, highlights what a former Information Commissioner of Canada identified as the systemic barriers and “technical arcana of bureaucracy” which “erode requesters’ right to timely access to information. This right is at risk of being totally obliterated because delays threaten to render the entire access regime irrelevant in our current information economy” (Legault, 2010, p. 2). Figure 2 provides a visual summary of the researcher’s access and ATIP request timelines.

## Accountability and Information Control: Government Transparency as “Public Myth”

In both cases, the original goal was to conduct qualitative research inside federal penitentiaries; when access was denied, we were each forced to find other ways to acquire information and access to key informants. One of us mobilized ATIP to acquire documents unavailable to the public, while the other did so to seek backstage government information that would reveal how her application for access was evaluated. These experiences prompt questions about government accountability and transparency, which Yeager (2012) describes as “a certain kind of public myth” (p. 169). Our experiences attempting to “excavate information” (Piché, 2012, p. 236) from the CSC reveal that the real burden of accountability lies not with the CSC to provide access to information, but with those seeking government information and materials to document and expose the barriers they face doing so. We suggest that the legislation that is designed to provide the Canadian public with opportunities to learn more about the inner workings of their government is conversely also used to maintain and legitimize secrecy in the interest of information control (Larsen & Walby, 2012). As Moore et al. (forthcoming) contend:

In contrast to other environments that have been diagnosed as transparency deficient (like hospitals or care facilities), increasing carceral transparency is cast as a threat to institutional security and society at large, thus necessitating its intensive management (Watson, 2015). . . carceral systems are accountable to a legislative mandate of security, correction, and reintegration that transposes dominant expectations of transparency, legitimizing a highly selective filtering of information and access. Herein lies the paradox of transparency: carceral governance favours performances of transparency that work to uphold a veil of secrecy.

There are a variety of techniques of opacity that government agencies engage to prevent journalists, researchers, and other members of the public from accessing requested information (Larsen & Walby, 2012). Our ATIP journeys far exceeded the 30-day statutory limit; indeed, given that the requested records were only disclosed due to pressure from OIC and OPC investigators, we agree with others who contend that the perpetual delay and postponement of access to records is a way to attempt to avoid disclosure (Hazell & Worthy, 2010; Luscombe & Walby, 2017). As this technique of opacity is especially common (Hazell & Worthy, 2010; Piché, 2012), there is little accountability when government agencies disregard this aspect of the ATI legislation. This point illustrates the contradictory nature of using ATI legislation to foster government accountability and transparency, where obfuscation and information gatekeeping create an “uneven form of accountability” which tends to create “secrecy through exemption and provide a source of rhetorical legitimacy for governments” (Larsen & Walby, 2012, p. 7). There is little to no recourse when subject to such delays; the requester must either abandon their request or pursue it through the OIC or OPC.

Our experiences also demonstrate the arduous process of brokering access. While negotiating access is part-and-parcel of requesting government information (Walby & Larsen, 2011b), it is nevertheless another mechanism through which governments can deflect responsibility while appearing to be accountable in what is “often times [a] chaotic process” (Walby & Luscombe, 2019, p. 2). As Luscombe et al. (2017) contend, “by brokering [access], FOI users are doing more than just invoking a legal right; they are also navigating the ‘wild’ complexities of legal argumentation, negotiation, precedence, and appeal” (p. 260). The first author spent months negotiating access to internal documents; she was asked several times to rephrase and narrow the scope of her request, which may expedite the process but may also cost access to important—hitherto unknown—information (Walby & Larsen, 2011a).

Another form of information control is evident in the final release of records. Indeed, the format in which a government department chooses to release records is significant. In some cases, the government department releases hard copies of documents, resulting in thousands or tens of thousands of pages of information, even though electronic copies were requested and are required by law to be produced (Luscombe & Walby, 2017). While the first author received electronic copies of the requested information, she received 11,000 pages of CSC documents in a single, unsearchable PDF file provided on a CD, which subsequently made analysis more difficult than it needed to be. Despite requesting information from the same government agency, the second author received paper copies of the requested material in the mail with long sections entirely redacted (there were also full sections omitted from and redacted in the first authors’ disclosure package). Luscombe and Walby (2017) suggest that providing material in unusable formats is a way for government agencies to control information flows. While redaction and omission are permitted by section 16 of the *Access to Information Act* and are essential in terms of maintaining anonymity, the degree to which the requested material is redacted certainly leaves the researcher guessing as to what content originally existed in the exempted space (Walby & Larsen, 2011b). In the final section, we explore what these techniques of opacity and attempts at information blockade and gatekeeping mean for the state of critical criminological scholarship in Canada.

## The Harms and Prejudices of Administrative Criminology in the Canadian Context

In 1986, Jock Young mapped a disturbing trend in the discipline of criminology toward an increasing research focus on the efficiency of the criminal justice system, which he dubbed “administrative criminology.” Administrative criminology has been described as decidedly atheoretical and depoliticized, subsequently lending itself to quick adoption by government researchers (Chunn & Menzies, 2006). While recognizing the value in some administrative criminological research, such as that which provides descriptive statistics about populations and practices, there are deep fault lines in the kinds of data administrative criminologists collect and make public. For example, neither Canadian policing services nor the CSC collects the kind of race-based data that are needed to ascertain and critique systemic racism in these fields (Millar & Owusu-Bempah, 2011). According to Hirschi (1993), this is because:

Academic criminology admits the existence of rival explanatory hypotheses. Administrative criminology is not so comfortable with the possibility that it may be barking up the wrong tree. . . Administrative criminology seems decidedly reluctant to raise questions about the current activities of its sponsors. But I am being too kind. The present report supports extension of current crime-control practices on the sole condition that they be adequately evaluated. (p. 349)

Hirschi’s point about evaluation hits close to home in Canada, where “the verity of the ‘programmers’ claims to ‘success’ are often ‘proven’ by dubious self-report questionnaire evidence from prisoners that a programme ‘works’—usually in terms of changing prisoners’ understanding of their offending behaviour” (Carlen, 2002, p. 120). In contradistinction, critical criminology is a branch of the discipline that examines the institutional and structural roots of crime and harm as originating in inequality and inequity rather than individual motivations. There are many different epistemological approaches that fall within the critical criminology paradigm, including, but not limited to feminism, postmodernism, convict criminology, and conflict theory. Critical criminologists analyze the ways in which oppression and discrimination in law and the penal field occur by way of class, gender, race, sexuality, ability, and other status and identity markers and prioritize consideration of institutional power dynamics and the material experiences of those who have come into conflict with the law, making access to carceral sites and actors key to their work.

The second author’s ATIP data reveal Hirschi’s (1993) point about the nature of administrative criminology in Canada, where external researchers are expected to conduct work that benefits the CSC, but without questioning their activities: “I am also curious about the researcher’s opinion on the contribution to the achievement of the Mission, the priorities of CSC and thoughts on the quality of the methodology and the benefits to CSC.” Requiring external research to directly benefit the CSC and its mission of administering punishment, formally described as “contribut[ing] to public safety by actively encouraging and assisting offenders to become law-abiding citizens, while exercising reasonable, safe, secure and humane control” (CSC, 2018) is a sure way to control information flows and to deny access to many, perhaps most, external scholars. Yet “as the custodians of information that belongs to Canadians, Parliament, the Information Commissioner and government must work with all stakeholders to achieve dynamic solutions that embrace democracy through the free flow of information” (Legault, 2010, p, 2). To oblige external researchers to produce research that CSC values is a demonstrable threat to Canadian citizens’ right to know. While exploratory research about people’s experiences working and living in carceral environments may not directly contribute to the CSC’s mandate regarding correctional punishment and rehabilitation, such scholarly inquiries are poised to ask the difficult but necessary questions about the institution’s policies and practices. This unreasonable methodological expectation subsequently operates as a gatekeeping measure that has little to do with the quality of the proposed project or its methodology, but rather with preventing critical minds from observing or hearing firsthand accounts of the prison’s inner workings.

Our attempts to conduct research in federal prisons and subsequent ATIP experiences highlight a troubling feature of administrative criminology, namely that government researchers control access not just to prisons as research sites, but also to staff and prisoners as essential sources of information about carceral interventions, practices, and the (inter)personal experiences that occur in these spaces. This degree of control over experiential voice or capital prevents staff, incarcerated people, and even those under community supervision from speaking to external researchers, especially critical prison scholars who problematize correctional decision-making, policies, and practices (Hannah-Moffat, 2011; Kilty, 2014).

In the ATIP data retrieved by the second author, the CSC “questioned if correctional officers would be willing to participate at all since one of the [research team] members . . . has had more critical views of CSC and more significantly of correctional officers,” a perplexing methodological assumption that suggests all correctional officers feel the same way about the practices they engage in. More ethically questionable is the fact that prior to encouraging the second author to request access to interview incarcerated people, the CSC Research Branch wanted to confirm that she would only be interviewing released prisoners who were no longer under CSC jurisdictional control—meaning, they were no longer on parole—a practice that seems poised to try to prevent people serving life-sentences from *ever* participating in external research. There is no obvious rationale as to why CSC maintains this research barrier, less an institutional commitment to the “epistemic injustice” (Fricker, 2007) of systematically excluding, silencing, and otherwise disqualifying criminalized people’s subjugated knowledges, which Foucault (1980, p. 84) contends we must foreground to challenge “the centralizing powers linked to the institution,” which is a core tenet of critical criminological research.

While recognizing that there must be an evaluation process and that it would be impossible to accept every application to conduct research in prison, what is especially problematic about barring external research access to prison sites is that this practice undermines and brings the Canadian Tri-Council research funding and peer-review process into disrepute. The Tri-council—made up of the Social Sciences and Humanities Research Council (SSHRC), the Canadian Institutes of Health Research (CIHR), and the Natural Sciences and Engineering Research Council (NSERC)—is the primary public funding body for academic research in Canada. Both authors’ projects were funded by the SSHRC; the first author’s project was funded by a Doctoral Fellowship while the second author’s project was funded by a 4-year Insight Grant, which means that both projects were positively peer-reviewed at the national level through the respective SSHRC assessment and evaluation processes as well as by the authors’ university REB prior to submitting their applications for research access to the CSC. When reputable scholars are prohibited from accessing public institutions to conduct research that is funded with public monies, the predominance of administrative criminology as the “received view” (Lincoln & Guba, 2003) in the discipline becomes painfully evident, adding further opacity to prison administration and oversight, which has long been shown to lack transparency (Arbour, 1996; Legault, 2010; Moore et al., forthcoming; Wright et al., 2015).

Accepting that the CSC’s external research application evaluation process is broken, as an alternative, we suggest that the Tri-council develop a community advisory board (CAB) to mediate between principal investigators and government agencies to facilitate access. The CAB should have members from the Tri-council, postsecondary-based researchers, regular community members, student representatives, and people with lived experience of the agency under investigation. Having a neutral, third-party body like a CAB would not only make the evaluation process more transparent, but it would also help to hold government agencies accountable in terms of permitting publicly funded research projects to move forward in a timely fashion. Timelier access would also help to ensure greater fiscal responsibility for researchers managing tri-council grants.

Another dilemma that our experiences help to reveal is one that Martel (2004) began to document 20 years ago, namely that government administrative criminologists have become emboldened to dismiss the value and significance of qualitative research, which they tend to do on dubious methodological grounds. van den Hoonaard (2014) problematizes this as a form of ethical and methodological colonization, writing, “qualitative research resembles a colonized country whose indigenous values and approaches are gradually being dissipated by the dominant, neopositivist model of research” (p. 173). Comments about the second author’s project found in the ATIP material demonstrate the use of a positivist approach to evaluate a qualitative project and how this epistemological misapplication of research rigor criteria effectively works to devalue the significance of qualitative research findings and subsequently to deny such scholars research access to carceral sites. For example, the CSC expressed concern that participants were to be given the opportunity to review and edit the anonymized transcript of their interview, a common qualitative research practice that preserves participant agency, helps to balance the power dynamics between researcher and participant, and ensures better quality research (Arnold et al., 2022; Grundy et al., 2003; Saldaña, 1998).

The CSC also suggested that there would be “bias in the types of offenders willing to participate in such a research study” and asked if there would “be good representation of offenders with ‘good’ storytelling experiences willing to volunteer?,” which more readily reflects an institutional fear and bias that incarcerated people will speak truth to power about the conditions of their confinement and state efforts to prohibit such voices from becoming public than it does any type of methodological bias inherent to the study’s design. Asking this question reveals institutional distrust in imprisoned people as reliable sources of information, which is a form of testimonial and thus epistemic injustice (Fricker, 2007) that undervalues and discredits their experiences. Such assertions not only work to delegitimize criminalized people’s accounts of incarceration, but they also debase their very capability to participate in research in meaningful ways. Given that administrative criminology largely involves evaluative research that decisively aims to show the benefits of correctional programs (Carlen, 2002; Hirschi, 1993), this comment also reveals the fundamental methodological flaws of CSC’s internal research which is biased in favor of presenting those interventions in a positive light.

Finally, the CSC suggested the second author shift her research design to instead “begin exploring ‘emotion culture’ in the community in spaces that are seen as critical to the reintegration process.” Illustrating an interesting pattern of attempting to divert research away from issues the Service finds too controversial, the second author (Kilty, 2014) documented a similar experience of being denied access to conduct research on self-harm in federal prisons for women, with the CSC suggesting that she change topics and research focus to explore how imprisoned women spend their leisure time in exchange for access. While the short email informing the second author that she was being denied access simply suggested that it was an inconducive time to conduct this research because the CSC was in a period of transition as they implemented new policy and operational requirements with respect to the SIUs and needle exchange, the ATIP data revealed that there were also concerns regarding “the level of disruption to operations, length of interviews, the security requirements to facilitate interviews with certain offenders.” This demonstrates that the counsel provided by staff at the Research Branch to change the initial proposal of interviewing staff and released prisoners was poor advice. It also shows the CSC’s failure to recognize the methodological value the project was offering in terms of documenting shifting views and feelings about such important policy transformations.

Our experiences of institutional opacity, gatekeeping, and physical research access denial reveal how administrative criminologists at the CSC effectively marginalize qualitative research and police criminological knowledge (Martel, 2004). We contend that this degree of “policing” is not only meant to disrupt information flows that prevent carceral transparency, but that it also reflects a dangerous methodological prejudice that effectively aims to discredit critical scholars by devaluing the significance of qualitative research. The irony of this kind of “prejudicial criminology” is that it fails to evaluate qualitative research on its own terms and problematically misapplies positivist evaluative criteria to interpretive research projects—an obvious methodological and evaluative error that was only brought to light through our use of ATIP methods.

## Conclusion

This article outlined how ATIP methods are being mobilized in Canadian critical prison scholarship and specifically described the tactics and strategies that the two authors used to acquire information and documents from the CSC when their applications for access to conduct qualitative research inside federal penitentiaries were denied. Notably, we discussed the techniques of opacity, information blockades, and institutional gatekeeping and protectionism we experienced and how these efforts combine to prevent the kind of government accountability and transparency that the Access to Information Act (1985) is supposed to ensure. We linked this lack of transparency to the predominance of administrative criminology in government research sectors, which we suggest aims to inhibit information flows in and out of carceral sites by preventing critical scholars from accessing carceral institutions to observe quotidian practices and interview prisoners and staff.

In taking up Piché’s (2011) argument that it is important to “go public” with one’s experiences and research dilemmas when using ATIP as a data collection method, we have tried to “contribute to a climate where information held by government agencies is disclosed” (p. 636). Notably, Piché (2011) mobilized his experiences of trying to broker access to engage in newsmaking criminology efforts (e.g., publishing media interventions) as a way to pressure prison authorities to release information. This is not part of our strategy with this article, although it is an aspect of the second author’s ongoing research program.^9^ By going public, we also contribute to the growing body of literature on access to information methods, particularly as it relates to the policing of criminological knowledge. While ATIP/FOI involve complex and lengthy processes, there is a demonstrable need for scholars to pursue these avenues of data collection, particularly with respect to information held by criminal justice and other law enforcement agencies, for without it we have fewer direct lines of sight into carceral institutions that will continue to be “black boxes.”

The pursuit of government transparency—in this case, prison transparency—is arduous at best (Moore et al., forthcoming), and it falls to researchers to document their experiences with the systemic flaws of different access to information regimes (Piché, 2011). Doing so importantly includes accessing backstage texts (as the second author did), including email correspondence, meeting minutes, and memorandums of understanding, to offer a unique perspective and to “conceptualize how government agencies work in action” (Walby & Larsen, 2011a, p. 39). While we were each denied physical access to conduct research in federal prisons, we were able (very slowly, and after a series of delays) to hold the CSC accountable by filing complaints with the Office of the Information Commissioner and the Office of the Privacy Commissioner to help us obtain the requested records. Given that most applications to conduct research in Canadian prisons are denied (Kilty, 2014; Martel, 2004), it is exceptionally important for the state of critical prison studies in this country that scholars embrace the use of ATIP/FOI as data collection methods to better understand the institutional and decision-making dynamics that shape the carceral environments that we are routinely prohibited from observing *in situ*.

## Notes

1. ATIP refers to federal Access to Information and Privacy requests while Freedom of Information (FOI) refers to provincial requests. The acronym ATI (access to information) tends to be a “universal” reference to any access to information requests.

2. In Canada, the term quasi-constitutional is used to describe laws that remain paramount even when newer statutes that contradict them are enacted. This is the reverse of the normal practice, under which newer laws trump contradictory provisions in older statutes.

3. This statement, made based on scholars’ published accounts of their experiences, is anecdotal because the CSC does not report the number of external applications to conduct research in prison that they receive. Future research should submit an ATIP request to solicit this kind of information and should consider surveying and/or interviewing post-secondary scholars about their experiences of attempting to gain access.

4. We must acknowledge that a small number of scholars do gain access to conduct research in prison. For example, there are currently teams from at least two Canadian universities that are conducting research inside. That said, access can sometimes involve research partnerships with and/or funding being provided by the CSC which means the researcher has fiscal obligations to the institution under investigation, an inherent conflict of interest. In such partnerships, the CSC shapes the research design, which must benefit and serve the CSC’s mandate (discussed further below).

5. The OIC’s final report revealed that my request sat idle at the CSC for 5 months, which is when the Service was pressured to work on it by the OIC investigator.

6. The ATI reference number for this request was A-2017-00397, which others may use to obtain copies of the acquired materials (Larsen & Piché, 2009).

7. I contacted the OPC rather than the OIC because the information sought is legally considered personal as it pertained to my application for research access.

8. As it was a privacy request, I cannot cite an ATIP reference number.

9. The second author is co-investigator on a seven-year SSHRC partnership grant entitled the Prison Transparency Project (PTP) (https://www.prisontransparencyproject.com) designed to examine cultures of carceral transparency across three countries—Canada, Spain, and Argentina. She presented this article at the Universidad Nacional del Litoral (Santa Fe, Argentina) and the Universidad de Buenos Aires (Argentina) in December 2023 with Argentinian prison authorities and correctional watchdogs present. With correctional watchdogs in all three countries as research partners, the PTP is investigating carceral research access via ATIP, interview, and survey methodologies. The PTP’s mandate is to produce academic papers, technical reports, and diverse media interventions that will engage extra-academic stakeholders to try to enhance carceral transparency by documenting information gatekeeping and access blockades.
